# Social Connectedness as Facilitators of Healthcare Access for Older Adults Living Alone with Disabilities

**DOI:** 10.1093/geroni/igaf122.2997

**Published:** 2025-12-31

**Authors:** Natalie Turner, Hyun-Jun Kim, Hailey Jung, Karen Fredriksen-Goldsen

**Affiliations:** University of Washington, Seattle, Washington, United States; University of Washington, Seattle, Washington, United States; University of Washington, Seattle, Washington, United States; University of Washington, Seattle, Washington, United States

## Abstract

Older adults with disabilities experience challenges accessing healthcare, resulting in preventable morbidity and mortality. Of particular concern is the disproportionate prevalence of living alone among people with disabilities – over 30% of people with disabilities live alone. While reflecting autonomy, living alone can contribute to the disablement process, social isolation, and increased healthcare needs. Using the Network Episode Model and data from the Health Equity and Intersectionality Study (n = 260), we examine the role of social connections as facilitators of health information and service navigation as well as individual and structural factors in predicting healthcare access measured by routine checkups, usual source of care, and having a personal doctor. We further estimate the interaction effects to examine if the relationships between these predictors and healthcare access differ by disability status and living arrangements. Logistic regression analyses, controlling for chronic conditions and sociodemographic characteristics, reveal that having more core confidants who provide support for health needs and share health-related information significantly predicts a higher likelihood of receiving routine check-ups, especially for older adults living alone with disabilities. Having a usual source of care is significantly associated with health literacy, with this relationship more pronounced among those living alone with disabilities. Having a personal doctor is positively associated with both health literacy and help-seeking behaviors regardless of disability status and living arrangements. Our findings inform the development of a conceptual model of the social dynamics that either facilitate or impede healthcare access among older adults, particularly those living alone with disabilities.

